# Assessing cost-effectiveness of HPV vaccines with decision analytic models: what are the distinct challenges of low- and middle-income countries? A protocol for a systematic review

**DOI:** 10.1186/s13643-015-0057-8

**Published:** 2015-05-12

**Authors:** Obinna I. Ekwunife, Andreas Gerber Grote, Christoph Mosch, James F. O’Mahony, Stefan K. Lhachimi

**Affiliations:** Cooperative Research Group for Evidence-Based Public Health, Department of Prevention and Evaluation, Leibniz Institute for Prevention Research and Epidemiology—BIPS, Bremen, Germany; Department of Clinical Pharmacy and Pharmacy Management, Nnamdi Azikiwe University, Awka, Nigeria; Institute for Quality and Efficiency in Health Care (IQWiG), Cologne, Germany; Institute for Research in Operative Medicine (IFOM), Universität Witten/Herdecke, Witten, Germany; Department of Health Policy and Management, School of Medicine, Trinity College Dublin, Dublin, Ireland; Institute for Public Health and Nursing Research—IPP, Health Sciences Bremen, University of Bremen, Bremen, Germany

**Keywords:** Uterine cervical neoplasm, Papillomavirus vaccines, Mass vaccination, Cost-effectiveness analysis, Low- and middle-income countries

## Abstract

**Background:**

Cervical cancer poses a huge health burden, both to developed and developing nations, making prevention and control strategies necessary. However, the challenges of designing and implementing prevention strategies differ for low- and middle-income countries (LMICs) as compared to countries with fully developed health care systems. Moreover, for many LMICs, much of the data needed for decision analytic modelling, such as prevalence, will most likely only be partly available or measured with much larger uncertainty. Lastly, imperfect implementation of human papillomavirus (HPV) vaccination may influence the effectiveness of cervical cancer prevention in unpredictable ways. This systematic review aims to assess how decision analytic modelling studies of HPV cost-effectiveness in LMICs accounted for the particular challenges faced in such countries. Specifically, the study will assess the following: (1) whether the existing literature on cost-effectiveness modelling of HPV vaccines acknowledges the distinct challenges of LMICs, (2) how these challenges were accommodated in the models, (3) whether certain parameters systemically exhibited large degrees of uncertainty due to lack of data and how influential were these parameters on model-based recommendations, and (4) whether the choice of modelling herd immunity influences model-based recommendations, especially when coverage of a HPV vaccination program is not optimal.

**Methods:**

We will conduct a systematic review to identify suitable studies from MEDLINE (via PubMed), EMBASE, NHS Economic Evaluation Database (NHS EED), EconLit, Web of Science, and CEA Registry. Searches will be conducted for studies of interest published since 2006. The searches will be supplemented by hand searching of the most relevant papers found in the search. Studies will be critically appraised using Consolidated Health Economic Evaluation Reporting Standards (CHEERS) statement checklist. We will undertake a descriptive, narrative, and interpretative synthesis of data to address the study objectives.

**Discussion:**

The proposed systematic review will assess how the cost-effectiveness studies of HPV vaccines accounted for the distinct challenges of LMICs. The gaps identified will expose areas for additional research as well as challenges that need to be accounted for in future modelling studies.

**Systematic review registration:**

PROSPERO CRD42015017870.

## Background

Cervical cancer poses one of the greatest challenges to women’s health. It is estimated that over a million women worldwide currently have cervical cancer, most of whom have not been diagnosed nor have access to treatment that could cure them or extend their survival [1]. The burden of cervical cancer is disproportionately borne by poorer countries. In 2012, 528,000 new cases of cervical cancer were diagnosed, and 266,000 women died of the disease, nearly 90 % of them in low- and middle-income countries (LMICs) [1]. It is expected that these numbers will double in the next 20 years due to aging and population growth [2].

The human papillomavirus (HPV) is the primary cause of cervical cancer and cervical intraepithelial neoplasia (CIN) [3]. HPV is typically transmitted in the cervix through microabrasions that may occur as a result of sexual intercourse [4]. Persistent infection with oncogenic strains of HPV causes cervical cancer [5]. Of the 40 HPV strains that affect the genital area, 15 of them are known to be oncogenic (types 16, 18, 31, 33, 35, 39, 45, 51, 52, 56, 58, 59, 68, 73, and 82) [6]. HPV infection has also been linked with other genital cancers (e.g., vaginal, vulvar, anal, and penile) as well as non-life-threatening diseases, such as genital warts [7]. HPV types 16 and 18 have been found to be responsible for about 70 % of all cervical cancer cases worldwide [8, 9]. In the remaining 30 % of cervical cancers, HPV types 31, 33, 35, 45, and 58 have all been implicated [10].

Cervical cancer can be prevented both by primary prevention through HPV vaccination and by secondary prevention through cervical cancer screening. Conventional cytology-based screening, combined with colposcopy and treatment of precursor lesions, has been the gold standard for the secondary prevention of cervical cancer. It has greatly reduced cervical cancer incidence and mortality from cervical cancer in many developed countries. Cytology-based screening is not as widespread in developing countries [10]. For instance, it is estimated that less than 5 % of women at risk of cervical cancer in sub-Saharan Africa have ever been screened [11].

Alternatives to cytology-based screening include visual inspection with acetic acid (VIA) and newer molecular testing for infection of the cervix with high-risk types of HPV DNA. VIA and HPV DNA testing have proven to be effective screening methods [12, 13]. HPV DNA testing has shown to be significantly more effective than VIA or cytology in reducing both cervical cancer precursors and cervical cancer [12, 13].

There currently are two HPV vaccines in widespread use. Both Cervarix® and Gardasil® offer protection against HPV 16 and 18, respectively, the two most oncogenic types [7]. Gardasil® also offers protection against HPV 6 and 11, which cause 90 % of genital warts [14]. The quadrivalent vaccine has also shown to protect against cancers of the anus, vagina, and vulva [14]. There is evidence indicating that the immune response against type 16 and 18 provides some cross-protection against types 45 and 31, both important in the aetiology of cervical cancer, thus potentially increasing the projected protection from vaccination to 75–80 % [10]. However, since prophylactic vaccination is not effective against infection from all 15 oncogenic HPV types, regular screening is still recommended among women that have received the vaccination [14].

### Challenges of prevention and control strategies in low- and middle-income countries

The World Health Organization’s (WHO) recommendation for comprehensive cervical cancer prevention and control strategy includes primary, secondary, and tertiary prevention activities [1]. Primary prevention involves HPV vaccination of girls (and boys if affordable) between 9 to 13 years. In secondary prevention, women 30 years of age or older are to be “screened-and-treated” with low-cost technology, e.g., VIA followed by cryotherapy or HPV testing for high-risk HPV type. For tertiary prevention, all women with invasive cancer at any age are to be treated with ablative surgery, radiotherapy, or chemotherapy with palliative care as necessary. The recommendation suggests that the three prevention components be planned and implemented in combination with a structured national approach to community education and mobilization strategies and a national monitoring and evaluation system [1].

LMICs potentially face a number of challenges in implementing such a cervical cancer prevention and control program. Existing health services may not have the capacity to accommodate additional interventions, and thus, extra cost will be incurred to set up such a program. Resource constraints may necessitate implementation of a program in phases rather than immediate implementation. Resource constraints could also require the introduction of preventive and treatment services only for certain regions rather than country-wide. Sections of the population may systematically avoid participation in such a program. Given these potential challenges, it is therefore important to examine if and how they are accounted for by decision analytic models of HPV vaccines; for example, do analyses adopt a different modelling structure or just change parameters for the constraints mentioned above?

### Data challenges in low- and middle-income countries

The data needed for modelling might be less readily available in LMICs or measured with much larger uncertainty. HPV-related outcome data in many LMICs may have poor resolution and are also often highly aggregated [15]. For example, incidence data of cervical cancer available from the WHO/Institut Català d’Oncologia Information Center on HPV and Cervical Cancer is stratified into the age groups 0–14, 15–44, 45–54, 55–64, and ≥65 years, and thus, simple natural history models may erroneously predict that 15-year-old women have the same cancer incidence as 44-year-old women, and this overestimates the proportion of cancers occurring in younger women and thus potentially biases estimates of vaccination cost-effectiveness [15]. These data challenges prompt the questions of how well they are recognized and overcome by the existing literature and which of these uncertain parameters have the greatest impact on the model outcomes. These are pertinent questions, and it is worthwhile examining how they have been handled in modelling studies of HPV vaccination.

### Model type and herd immunity

There are three types of models that have been employed in cost-effectiveness analyses of HPV vaccination: static models, transmission dynamic models, and hybrid models combining features of both static and dynamic models [7]. A static model typically tracks progression of HPV disease for a single cohort over an expected lifetime [16]. Transmission dynamic models have the advantage of accounting for both the direct and indirect (i.e., herd immunity) benefits of vaccination in the population [16]. Thus, dynamic models account for the immunity that occurs when HPV vaccination of a significant portion of the population (or herd) provides a measure of protection for individuals who have not been vaccinated. A hybrid model is a combination of a cohort model and a dynamic model. It corrects the invariant incidence probability in a cohort model to a dynamic probability and thus does not ignore the indirect benefits of herd immunity for the cohort being simulated [16]. However, herd immunity depends on the rate of vaccine coverage. As stated previously, this coverage may vary dramatically in LMICs, both across time or across populations. It is therefore relevant to ask how this is accounted for in HPV vaccine cost-effectiveness studies.

### Study rationale and objectives

Appraising the introduction of HPV vaccines requires estimation of the avertable disease burden, cost-effectiveness of the vaccine compared with alternative uses of the resources, affordability of the vaccine, feasibility of achieving high coverage, likelihood of public acceptability, and political support for vaccination [17]. The lack of data on the long-term effectiveness of HPV vaccination has prompted the development of various decision analytic models to guide policy makers by projecting the long-term epidemiologic and economic consequences of alternative vaccination policies [16]. For such analyses to provide a reliable guide to policy development and implementation, they should reflect the LMIC-specific challenges described above.

Two systematic reviews of cost-effectiveness analyses of HPV vaccination in LMICs have been published previously [18, 19]. These reviews discussed the cost-effectiveness estimates and investigated how they are affected by model characteristics and underlying assumptions in general. The focus of our proposed systematic review is to examine how the modelling studies accounted for the challenges specific to low- and middle-income countries. The review seeks to answer the following questions:

- Does the existing literature on cost-effectiveness modelling of HPV vaccine acknowledge the particular challenges facing LMICs?
- How were particular challenges accommodated in the models, e.g., through a different model structure or just by varying parameters?
- Is the uncertainty among the less readily available essential data/parameters regarding LMICs so large that the model-based recommendation is affected?
- Does the choice of modelling herd immunity influence model-based recommendations, and in particular, does imperfect HPV vaccination coverage influence model-based recommendations?

## Methods/design

### Protocol

This protocol adheres to the Preferred Reporting Items in Systematic Reviews and Meta-analyses (PRISMA) statement [20]*.* The protocol is registered in the International Prospective Register of Systematic Reviews (PROSPERO) CRD42015017870.

### Eligibility criteria

Inclusion criteria are as follows:

- Studies based on decision analytical models of HPV vaccination;
- Studies that considered the cost-effectiveness of HPV vaccination and reported the additional cost and additional health effects in terms of life years gained (LYGs), quality-adjusted life years (QALYs), or disability-adjusted life year (DALYs);
- LMICs as identified by the World Bank classification of income groups [21];
- Both single- and multi-country studies;
- The review will include both original research papers and reviews (inclusion of the latter is to ensure that no original study was missed);
- Studies to be included in the review could be published in any language;
- Studies published since 2006.

### Information sources

We will search MEDLINE (via PubMed), EMBASE, NHS Economic Evaluation Database (NHS EED), EconLit, Web of Science, and Tufts CEA Registry. For existing systematic reviews, we will search Cochrane Reviews, Cochrane Database of Abstracts of Reviews of Effects (DARE), and Cochrane Health Technology Assessment Databases. Reviews will be included to reduce the possibility of missing an individual study.

### Search strategy

A search strategy will be developed for each of the databases. The “Appendix” section provides details of our planned bibliographic database search strategies for MEDLINE (via PubMed), EMBASE, CINAHL, Cochrane Reviews/Cochrane DARE/NHS EED, EconLit, Web of Science, and CEA Registry. The reference lists of all included and relevant articles identified during the search will be reviewed to identify further studies that were missed. Furthermore, we will use the PubMed “related articles” feature. Hand searching of a selection of relevant journals will be conducted as advised by experts in economic evaluation.

### Study selection

Titles and abstracts will be screened for inclusion independently by two of the reviewers using the eligibility criteria. Opinion of a third reviewer will be sought to arrive at a consensus in case of disagreement on a study for inclusion.

### Data extraction

Data will be extracted independently by two of the reviewers from included studies using a predefined data extraction spreadsheet (Table 1). Data to be extracted will be arranged into the following classes: model characteristics, base-case assumptions, results, sensitivity/uncertainty analyses, data sources, and miscellaneous (conflict of interests and factors not considered). Cost presented in different currencies will be adjusted to 2013 value using consumer price index. Afterwards, cost data will be converted to international dollar units using purchasing power parities (PPPs). Authors of various studies may be contacted to clarify methods and results if the need arises.

**Table 1:** Relevant data extraction information

Data extraction category	Specific information to extract
*A. Model characteristics*	
Model	Author(s), year of publication, model design (static model, dynamic model, or hybrid model)
Perspective	Providers, patients, or societal perspective
Benefits (QALY, DALY, YLS)	Measured in quality-adjusted life years (QALYs), disability-adjusted life years (DALY), years of life saved (YLS)
*B. Base-case assumptions*	
Current routine practice efficacy	Efficacy of the current screening practice (cytology-based screening, DNA screening, or visual inspection with acetic acid) used as the comparator
Screening age/screening interval	Age at which women commence screening and screening interval
Vaccine coverage in target groups	Current HPV vaccination coverage
Age for vaccination	Age group eligible to receive HPV vaccination
Estimated effective coverage	HPV vaccination coverage and the rationale for the assumption
Screening compliance	Estimate used for comparison with current practice
Sensitivity/specificity of the screening	Sensitivity/specificity estimate for the screening methods
Duration of vaccine protection	Total length of time HPV vaccination is assumed to protect the recipient from acquiring infection
Cost of vaccine per three doses (and booster if included)	Market cost or subsidized cost. Other cost associated with vaccination, e.g., freight, storage, and program cost. School-based-delivery cost or health-facility-based delivery cost
Discounting rate	Discounting rate used to adjust for time preference for money
*C. Results*	
Incremental cost-effectiveness ratio (ICER)	The most cost-effective protocol compared with the second best protocol
Year based for currency value	The year which the analysis was conducted
Adjusted ICER (to 2014)	Adjusted ICER to reflect 2014 value
*Data sources*	
*Source(s)*	Data source used to derive estimates of HPV-related epidemiologic outcomes, e.g., HPV (type-specific) prevalence, cervical cancer incidence, probability of HPV transmission given a sexual partnership, and crude mortality from cervical cancer.
*D. Sensitivity and uncertainty*	
Sensitivity analysis	Parameters that had the highest effect on model-based recommendation.
Uncertainty analysis	The contribution of individual parameters on overall uncertainty (when reported).
*E. Miscellaneous*	
Funding and conflict of interest	Funding for the study and the role of the funder in the study. Possible conflict of interest declared by the author
Factors not taken into account	How did model acknowledge and account for special challenges of the LMIC? Choice of modelling herd immunity

### Risk of bias and data synthesis

One of the investigators will assess the validity of included studies using the Consolidated Health Economic Evaluation Reporting Standards (CHEERS) statement [22]. Details of the CHEERS statement are summarized in Table 2. Descriptive, narrative, and interpretative synthesis of data will be undertaken to address the study objectives [23]. The WHO Commission on Macroeconomics and Health will be used to determine the thresholds of cost-effectiveness such that an intervention will be considered “very cost effective” and “cost effective” when its incremental cost-effectiveness ratio is less than the gross domestic product (GDP) per capita and less than three times GDP per capita, respectively [23].

**Table 2:** CHEERS statement for checking the validity of included studies [22]

Selection/item	Item no.	Recommendation
*Title and abstract*		
Title	1	Identify the study as an economic evaluation, or use more specific terms such as “cost-effectiveness analysis” and describe the interventions compared.
Abstract	2	Provide a structured summary of objectives, perspective, setting, methods (including study design and inputs), results (including base-case and uncertainty analyses), and conclusions.
*Introduction*		
Background and objectives	3	Provide an explicit statement of the broader context for the study. Present the study question and its relevance for health policy or practice decisions.
*Methods*		
Target population and subgroups	4	Describe characteristics of the base-case population and subgroups analyzed including why they were chosen.
Setting and location	5	State relevant aspects of the system(s) in which the decision(s) need(s) to be made.
Study perspective	6	Describe the perspective of the study and relate this to the costs being evaluated.
Comparators	7	Describe the interventions or strategies being compared and state why they were chosen.
Time horizon	8	State the time horizon(s) over which costs and consequences are being evaluated and say why appropriate.
Discount rate	9	Report the choice of discount rate(s) used for costs and outcomes and say why appropriate.
Choice of health outcomes	10	Describe what outcomes were used as the measure(s) of benefit in the evaluation and their relevance for the type of analysis performed.
Measurement of effectiveness	11a	*Single study-based estimates*: describe fully the design features of the single effectiveness study and why the single study was a sufficient source of clinical effectiveness data.
	11b	*Synthesis-based estimates*: describe fully the methods used for the identification of included studies and synthesis of clinical effectiveness data.
Measurement and valuation of preference-based outcomes	12	If applicable, describe the population and methods used to elicit preference outcomes.
Estimating resources and costs	13a	*Single study-based economic evaluation*: describe approaches used to estimate resource use associated with the alternative interventions. Describe primary or secondary research methods for valuing each resource item in terms of its unit cost. Describe any adjustments made to approximate to opportunity costs.
	13b	*Model-based economic evaluation*: describe approaches and data sources used to estimate resource use associated with model health states. Describe primary or secondary research methods for valuing each resource item in terms of its unit cost. Describe any adjustments made to approximate to opportunity costs.
Currency, price date, and conversion	14	Report the dates of the estimated resource quantities and unit costs. Describe methods for adjusting estimated unit costs to the year of reported costs if necessary. Describe methods for converting costs into a common currency base and the exchange rate.
Choice of mode	15	Describe and give reasons for the specific type of decision analytic model used. Providing a figure to show model structure is strongly recommended.
Assumptions	16	Describe all structural or other assumptions underpinning the decision analytic model.
Analytic methods	17	Describe all analytic methods supporting the evaluation. This could include methods for dealing with skewed, missing, or censored data; extrapolation methods; methods for pooling data; approaches to validate or make adjustments (e.g., half-cycle corrections) to a model; and methods for handling population heterogeneity and uncertainty.
*Results*		
Study parameters	18	Report the values, ranges, references, and, if used, probability distributions for all parameters. Report reasons or sources for distributions used to represent uncertainty where appropriate. Providing a table to show the input values is strongly recommended.
Incremental costs and outcomes	19	For each intervention, report mean values for the main categories of estimated costs and outcomes of interest, as well as mean differences between the comparator groups. If applicable, report incremental cost-effectiveness ratios.
Characterizing uncertainty	20a	*Single study-based economic evaluation*: describe the effects of sampling uncertainty for estimated incremental cost, incremental effectiveness, and incremental cost-effectiveness, together with the impact of methodological assumptions (such as discount rate, study perspective).
	20b	*Model-based economic evaluation*: describe the effects on the results of uncertainty for all input parameters and uncertainty related to the structure of the model and assumptions
*Discussion*		
Study findings, limitations, generalizability, and current knowledge	22	Summarize key study findings and describe how they support the conclusion reached. Discuss limitation and the generalizability of the findings and how the findings fit with current knowledge.
*Others*		
Source of funding	23	Describe how the study was funded and the role of the funder in the identification, design, conduct, and reporting of the analysis. Describe other non-monetary sources of support.
Conflicts of interest	24	Describe any potential for conflict of interest among study contributors in accordance with journal policy. In the absence of a journal policy, we recommend authors comply with International Committee of Medical Journal Editors’ recommendations.

## Discussion

This protocol describes a systematic review for studies on cost-effectiveness of prophylactic HPV vaccination in LMICs. The aim is to assess how cost-effectiveness studies of HPV vaccine accounted for the individual challenges of low- and middle-income countries. The gaps identified in this systematic review will expose areas for additional research as well as challenges that need to be accounted for in future modelling studies. The review will also present current data on cost-effectiveness of HPV vaccines in LMICs as newer studies have been carried out since the publication of the last reviews.

## Appendix: Details of electronic bibliographic database search strategies

### MEDLINE (via PubMed)

(“Human papillomavirus 6” [mesh] OR “Human papillomavirus 16” [mesh] OR “Human papillomavirus 18” [mesh] OR “Human papillomavirus 31” [mesh] OR Alphapapillomavirus [mesh] OR Papillomavirus* [tiab] OR human papilloma* [tiab] OR HPV [tiab] OR “Papillomavirus Infections” [mesh] OR Papillomaviridae [mesh] OR Papillomavirid* [tiab] OR Uterine Cervical Neoplasms [mesh] OR cervix cancer* [tiab] OR cervix carcinom* [tiab] OR cervix malignan* [tiab] OR cervix neoplas* [tiab] OR cervix tumor* [tiab] OR cervical cancer* [tiab] OR cervical carcinom* [tiab] OR cervical malignan* [tiab] OR cervical neoplas* [tiab] OR cervical tumor* [tiab] OR Cervical Intraepithelial Neoplasia [mesh] OR Cervical Intraepithelial Neoplasia [tiab] OR CIN [tiab])

AND

(Vaccination [mesh] OR mass vaccination [mesh] OR Papillomavirus Vaccines [mesh] OR vaccin* [tiab] OR immunization [mesh] OR Immunization Programs [mesh] OR immuni* [tiab] OR “human papillomavirus vaccine L1, type 6,11,16,18” [Supplementary Concept] OR “human papillomavirus vaccine, L1 type 16, 18” [Supplementary Concept] OR Gardasil [tiab] OR Cervarix [tiab])

AND

(“2006/01/01”[dp] : “3000”[dp])

AND

(Economics [mesh] OR Quality of Life [mesh:NoExp] OR Value of Life [mesh:NoExp] OR Quality-Adjusted Life Years [mesh:NoExp] OR Models, Economic [mesh:NoExp] OR Markov Chains [mesh:NoExp] OR Monte Carlo Method [mesh:NoExp] OR Decision trees [mesh:NoExp] OR economic* [tiab] OR cost* [tiab] OR costing* [tiab] OR costly [tiab] OR costed [tiab] OR price* [tiab] OR pricing* [tiab] OR pharmacoeconomic* [tiab] OR pharmaco-economic* [tiab] OR budget* [tiab] OR expenditure* [tiab] OR (value [tiab] AND (money [tiab] OR monetary [tiab])) OR fee [tiab] OR fees [tiab] OR quality of life [tiab] OR qol* [tiab] OR hrqol* [tiab] OR quality adjusted life year* [tiab] OR qaly* [tiab] OR cba [tiab] OR cea [tiab] OR cua [tiab] OR utilit* [tiab] OR markov* [tiab] OR monte carlo [tiab] OR (decision [tiab] AND (tree* [tiab] OR analys* [tiab] OR model* [tiab])) OR ((clinical [tiab] OR critical [tiab] OR patient [tiab]) AND (path* [tiab] OR pathway* [tiab])) OR (managed [tiab] AND (care [tiab] OR network* [tiab])))

### (just) EMBASE

(‘Human papillomavirus type 6’/exp OR ‘Human papillomavirus type 16’/exp OR ‘Human papillomavirus type 18’/exp OR ‘Human papillomavirus type 31’/exp OR ‘Alphapapillomavirus’/exp OR ‘Papillomavirus Infection’/exp OR ‘Papilloma virus’/exp OR ‘uterine cervix cancer’/exp OR (Papillomavirus* OR human papilloma* OR HPV OR Papillomavirid*):ab,ti OR ((cervix OR cervical) NEAR/3 (cancer* OR carcinoma* OR malignanc* OR neoplasm* OR tumor*)):ab,ti OR ‘uterine cervix carcinoma in situ’/exp OR (“Cervical Intraepithelial Neoplasia” OR CIN):ab,ti)

AND

(‘Immunization’/exp OR ‘Cancer immunization’/exp OR ‘mass immunization’/exp OR ‘Vaccination’/exp OR ‘Wart virus vaccine’/exp OR (vaccin* OR immuni* OR Gardasil OR Cervarix):ab,ti OR ‘preventive health service’/exp)

AND

[1-1-2006]/sd NOT [1-1-3000]/sd

AND

(‘health economics’/exp OR ‘health care cost’/exp OR ‘quality of life’/exp OR ‘quality adjusted life year’/exp OR ‘Monte Carlo method’/exp OR ‘decision tree’/exp OR (economic* OR cost* OR costing* OR costly OR costed OR price* OR pricing* OR pharmacoeconomic* OR budget* OR expenditure* OR fee OR fees OR ‘quality of life’ OR qol* OR hrqol* OR qaly* OR CBA OR CEA OR CUA OR utilit* OR markov*):ab,ti OR (pharmaco NEXT/1 economic*):ab,ti OR (value NEAR/1 (money OR monetary)):ab,ti OR (‘quality adjusted life’ NEXT/1 year*):ab,ti OR (monte NEXT/1 carlo):ab,ti OR (decision NEXT/3 (tree* OR analys* OR model*)):ab,ti OR ((clinical OR critical OR patient) NEXT/1 (path* OR pathway*)):ab,ti OR (managed NEXT/3 (care OR network*)):ab,ti)

### EconLit and CINAHL (via EBSCO)

MH Papilloma OR MH Papillomaviruses OR MH “papillomavirus infections” OR AB (papilloma OR papillomavirus* OR HPV) OR MH “cervix neoplasms” OR MH “uterine neoplasms” OR AB ((cancer* OR carcinoma* OR malignanc* OR neoplasm* OR tumor*) N3 (uterine OR cervix OR cervical)) OR MH “Cervical Intraepithelial Neoplasia” OR AB CIN AND MH “papillomavirus vaccine” OR MH immunization OR MH “Immunization Programs” OR AB (immuni?ation OR vaccin* OR gardasil OR cervarix) AND PD (2006 OR 2007 OR 2008 OR 2009 OR 201*) AND MH Health Resource Allocation OR MH Health Care Delivery OR MH Health Care Costs OR MH Economic Value of Life OR MH Quality of Life OR MH Quality-Adjusted Life Years OR MH Decision Trees OR AB (economic* OR cost* OR costing* OR costly OR costed OR pri?e* OR pri?ing* OR pharmacoeconomic* OR budget* OR expenditure* OR fee OR fees OR “quality of life” OR qol* OR hrqol* OR qaly* OR CBA OR CEA OR CUA OR utilit* OR markov*) OR AB ((pharmaco N1 economic*) OR (value N1 (money OR monetary)) OR (“quality adjusted life” N1 year*) OR (monte N1 carlo) OR (decision N3 (tree* OR analys* OR model*)) OR ((clinical OR critical OR patient) N1 (path* OR pathway*)) OR (managed N3 (care OR network*)))

### Cochrane Reviews/Cochrane DARE/NHS EED/HTA database

MeSH descriptor: [Human papillomavirus 6] explode all trees OR MeSH descriptor: [Human papillomavirus 16] explode all trees OR MeSH descriptor: [Human papillomavirus 18] explode all trees OR MeSH descriptor: [Human papillomavirus 31] explode all trees OR MeSH descriptor: [Alphapapillomavirus] explode all trees OR MeSH descriptor: [Papillomaviridae] explode all trees OR MeSH descriptor: [Papillomavirus Infections] explode all trees OR MeSH descriptor: [Uterine Cervical Neoplasms] explode all trees OR MeSH descriptor: [Uterine Neoplasms] explode all trees OR MeSH descriptor: [Cervical Intraepithelial Neoplasia] explode all trees OR (Papillomavirus* OR human papilloma* OR HPV OR Papillomavirid* OR ((cervix OR cervical) NEAR/3 (cancer* OR carcinoma* OR malignanc* OR neoplasm* OR tumor*)) OR “Cervical Intraepithelial Neoplasia” OR CIN):ti,ab,kw

AND

MeSH descriptor: [Papillomavirus Vaccines] explode all trees OR MeSH descriptor: [Immunization] explode all trees OR MeSH descriptor: [Vaccination] explode all trees OR MeSH descriptor: [Mass Vaccination] explode all trees OR MeSH descriptor: [Immunization Programs] explode all trees OR (vaccin* OR immuni* OR Gardasil OR Cervarix):ti,ab,kw

AND

MeSH descriptor: [Economics] explode all trees OR MeSH descriptor: [Economics, Medical] explode all trees OR MeSH descriptor: [Economics, Pharmaceutical] explode all trees OR MeSH descriptor: [Health Care Economics and Organizations] explode all trees OR MeSH descriptor: [Health Care Costs] explode all trees OR MeSH descriptor: [quality of life] explode all trees OR MeSH descriptor: [value of life] explode all trees OR MeSH descriptor: [Quality-Adjusted Life Years] explode all trees OR MeSH descriptor: [Markov Chains] explode all trees OR MeSH descriptor: [Monte Carlo method] explode all trees OR MeSH descriptor: [decision trees] explode all trees OR (economic* OR cost* OR costing* OR costly OR costed OR price* OR pricing* OR pharmacoeconomic* OR budget* OR expenditure* OR fee OR fees OR ‘quality of life’ OR qol* OR hrqol* OR qaly* OR CBA OR CEA OR CUA OR utilit* OR markov* OR (pharmaco NEXT/1 economic*) OR (value NEAR/1 (money OR monetary)) OR (‘quality adjusted life’ NEXT/1 year*) OR (monte NEXT/1 carlo) OR (decision NEXT/3 (tree* OR analys* OR model*)) OR ((clinical OR critical OR patient) NEXT/1 (path* OR pathway*)) OR (managed NEXT/3 (care OR network*))):ti,ab,kw

AND

Limit Publication Date: 01-1-2006 till now

### Web of Science (Core Collection)

TS = (((cervix OR cervical) NEAR/3 (cancer* OR carcinoma* OR malignanc* OR neoplasm* OR tumor*)) OR Papillomavirus* OR human papilloma* OR HPV OR Papillomavirid* OR “Cervical Intraepithelial Neoplasia” OR CIN)

AND

TS = (vaccin* OR immuni* OR Gardasil OR Cervarix)

AND

PY = (2006–2015) AND TS = (health econom* OR health care cost* OR Monte Carlo OR economic* OR cost* OR costing* OR costly OR costed OR pri?e* OR pri?ing* OR pharmacoeconomic* OR pharmaco economic* OR budget* OR expenditure* OR fee OR fees OR quality of life OR quality adjusted life year* OR qol* OR hrqol* OR qaly* OR CBA OR CEA OR CUA OR utilit* OR markov* OR (value NEAR/1 (money OR monetary OR life)) OR (decision NEXT/3 (tree* OR analys* OR model*)) OR ((clinical OR critical OR patient) NEAR/1 (path* OR pathway*)) OR (managed NEAR/3 (care OR network*)))

### CEA Registry

Basic/manual search by the following keywords (published 2006–2015): Papilloma, Papillomavirus, Papillomaviridae, HPV 74, cervix, cervical, uterine, Gardasil and Cervari

## Abbreviations

CHEERS: Consolidated Health Economic Evaluation Reporting Standards
CIN: Cervical intraepithelial neoplasia
DALYs: Disability-adjusted life years
GDP: Gross domestic product
HPV: Human papillomavirus
LMICs: Low- and middle-income countries
LYGs: Life years gained
NHS EED: NHS Economic Evaluation Database
PRISMA: Preferred Reporting Items in Systematic Reviews and Meta-analyses
VIA: Visual inspection with acetic acid
WHO: World Health Organization
